# Making Single Mothers Matter: Reflections on the Vulnerability and Agency of Displaced Persons in Postwar Occupied Austria and Beyond

**DOI:** 10.1177/01979183231218972

**Published:** 2024-01-15

**Authors:** Franziska M. Lamp-Miechowiecki

**Affiliations:** Department of Contemporary History, 27258 University of Vienna, Vienna, Austria

**Keywords:** International Refugee Organization (IRO), family displacement, displaced persons

## Abstract

This article explores the experiences of displaced women and their children in occupied postwar Austria by focusing both on the assistance provided to them by relief workers in displaced persons (DP) camps and on the displaced women's role as persons in charge of planning their own and their children's futures. In doing so, it sheds light on the care infrastructure for a particularly vulnerable group inside Austria's postwar refugee camps — single mothers and those children within their households — while also highlighting the agency of these women in navigating the migration process. The author of this paper argues that studying the situation of displaced single mothers enables us to understand displacement as a process that both fostered a dependence on institutional structures and at the same time created the imperative to develop specific strategies to negotiate one's chances of emigration, such as the use of social networks or the negotiation of citizenship. The main arguments of this paper are embedded in a close analysis of two DP camps in postwar Austria — Kapfenberg in Styria and Feffernitz in Carinthia. A combined examination of reports written by relief workers employed in these camps and two case studies of families that tried to emigrate and leave their camp lives behind allows the author to reflect on dimensions of vulnerability and agency — both of which were characteristic of the postwar experience of so many displaced people in Europe, especially those cast in maternal roles.

## Introduction

“What ‘family’ life can exist in such conditions?” asked Clare McMurray, a staff member of the Save the Children Fund (SCF) working in occupied Austria, in her report about the postwar situation in Kapfenberg, a town that was home to many so-called “displaced persons (DP)” camps (McMurray 1948, 2). And, yet the conditions there and elsewhere in camps for the uprooted victims of the Second World War and the Holocaust did support preexisting and growing families of various shapes and sizes. The situation of nursing mothers, widows, and single mothers with young children proved to be a particular challenge for the management of postwar relief and resettlement measures. Displaced mothers and their children were in a very vulnerable and unfavorable position. Not only did they have to take care of their children's health and nutrition in the camps, they also had to face gender hierarchies within the administration of emigration (Fitzpatrick 2022, 286, 295; Kevin and Agutter 2017, 554–555).

Despite attention paid to specific programs for the resettlement of single displaced women (Kay 2003, 153; Kevin and Agutter 2017; Dellios 2018), historians have not explored the unique circumstances surrounding displaced unmarried mothers of young children and how they negotiated with institutions which planned and implemented a variety of programs to assist them. This paper, which stems from the author's larger research project,^1^ visualizes the gender hierarchies that shaped the postwar displacement experience. In this context, examining the postwar reality of displaced, single mothers brings into sharper focus what we know about relief and resettlement in postwar Central Europe. Individual stories brought to life over the next few pages demonstrate that the experiences of displaced single mothers can best be explored through an approach that considers practices at the institutional level such as humanitarian attempts to improve the specific vulnerable position of women in DP camps, and the strategies of the displaced mothers themselves to negotiate their emigration. The paper pays specific attention to the situation of displaced single mothers^2^ transiting through postwar Austria, which until now has been neglected in research on postwar displacement in Europe.

This paper considers some of the many questions that arise specifically in connection with displaced single-mother families and which remain largely unanswered despite the rich historiography that has developed around the post-Second World War daily life in the DP camps. These include: how did caretakers help and care for DP mothers and their children? What was their attitude toward displaced mothers? What strategies did vulnerable DP populations like single mothers use to improve their chances of fulfilling their desires to emigrate? To what extent can we use existing sources to outline both the sensitive situations faced by families and the options they employed for navigating within seemingly complex parameters? The author of this paper seeks to answer some of these questions by using an innovative source base that forms the center of the following pages.

## Displacement in Postwar Austria

The immediate postwar period was characterized by the displacement of millions of people across the European continent and, in fact, around the world. Managing these migration and refugee movements soon became one of the most challenging tasks facing relief workers. In April 1945, the writer and journalist George Orwell, a notable observer of postwar Europe, traveled as a correspondent through war-torn Germany and Austria. He wrote compellingly about the catastrophic situation and the most urgent problems faced by displaced people in Europe: “It is not easy to find accommodation for these vast numbers of people, which include children born in captivity” (Orwell 1945, 62). In May 1945, he described “the encampments of the Displaced Persons, some of them sharing barracks with derelict German soldiers, while others have seized railway trains and are living in the carriages” (Orwell 1945, 89). Shortly after the war, around 1.65 million uprooted people lived in Austria (Olah 1964, 1). Among them were so-called “displaced persons” (DPs) — most of whom had either been forced to work for the National Socialist regime or were interned in concentration camps during the war — as well as German-speaking expellees, so-called “ethnic German”^3^ refugees, who could no longer return to their homes in South-East Central Europe. Together they now found themselves in need of help.

The term “DP” was used from 1944 in order to describe those people that had been brought to Austria by force during the war (Strobl and Hagen 2022, 37). However, the legal status of DPs and the corresponding eligibility for care and support from organizations like the International Refugee Organization (IRO), the successor organization of the United Nations Relief and Rehabilitation Administration (UNRRA), did not apply to “ethnic Germans” (Stieber 1997, 27–28). To adequately describe the situation of all the above-mentioned “groups”^4^ of forced migrants, this paper therefore uses the term “displaced people.”

Displaced people stood at the center of various policies on how to organize their accommodation in postwar Austria as well as their movement toward other potential receiving states. Many different institutional and national actors were involved in this process, among them the Allied governments and several international organizations — the most important of them being the UNRRA (later IRO). Furthermore, key for determining the guidelines for the onward movement of these displaced people from Europe were the policies of states which emerged and reemerged in Central and Eastern Europe, as well as the interests of potential refugee host states like Canada, Australia, or the United States (Stieber 1997, 287–290; Huhn 2021, 16–20). These three countries were the preferred destinations for emigrants from Austria, followed by Israel, France, the United Kingdom, and Argentina (Stieber 1997, 290–291). After a period in which the Allies and UNRRA enforced repatriation from Allied-occupied Germany and Austria to the former home countries of DPs and refugees the policy shifted to implementing emigration as another option when the IRO came to be in charge (Höschler 2020, 236). Hence, postwar Austria and Germany became nodes of transit for an exceedingly diverse group of people with different persecution histories and migration biographies. They were housed in hundreds of refugee camps all over the country where they were provided with a place to sleep, and access to health care, as well as information about emigration (Olah 1964, 2; Bacher and Perzi 2017, 198). A 1964 report by the Austrian Ministry of the Interior mentioned 300 different refugee camps in Austria (Olah 1964, 2). However, not all displaced people lived in camps. Many were housed in private quarters, often receiving little or no help from international welfare organizations, as this statement by British relief worker McMurray exemplifies: “I have had endless visits from DP's [*sic*] out of camp, requesting for clothing. It has been so hard to turn them away as so many have no help at all and are very badly off” (McMurray 1948, 8).

Although the various DP camps in postwar Austria housed very heterogeneous groups of people, most research on postwar displacement in Austria and Germany has applied a “single frame” (Zahra 2010, 192), focusing either on DPs (and here mainly on Jewish or Polish DPs) or on “ethnic Germans.” This segmentation in research can be attributed to the very varied persecution experiences of Jewish DPs and members of German-speaking minorities, which also resulted in a different treatment by the Allies and organizations like the IRO. When they arrived in occupied Austria, displaced people were enmeshed in complex situations. Jewish survivors of the Holocaust, other displaced people of various nationalities, and German-speaking refugees, found themselves in postwar Austria. Most of the latter had been forced to leave their homes in South-East Central Europe at the end of the war or had fled in fear of persecution by the Soviets and newly forming governments in Czechoslovakia, Poland, and Yugoslavia. Despite the different contexts, this paper argues, however, that a non-“compartmentalized” (Panagiotidis 2020, 174) approach allows new insights into the mechanisms of the post-Second World War “migration regime” (Rass and Wolff 2018, 19). It therefore examines the effects of forced migration on people with diverse backgrounds, revealing dimensions of both agency and discrimination regarding displaced, unmarried mothers. While important works exist on the specific situation of single mothers who were resettled in Australia (Williams 2014; Kevin and Agutter 2017; Stroja 2017), as well as studies looking into the role of traditional gender norms in DP camps in Europe (Feinstein 2006; Schlichting 2014; Zahra 2011; Nowak 2019; Königseder and Wetzel 2020) and during the resettlement process (Balint 2021; Fitzpatrick 2022), the historiography on refugee camps in postwar Austria still lacks a gender-based focus on the experiences of displacement and care as well as the facilitation of emigration and resettlement of displaced mothers.

## Sources on Vulnerability and Agency

This paper outlines dimensions of personal agency and vulnerability of displaced single mothers. For this purpose, two central source corpora were examined. First, reports by relief workers active in organizations like the SCF, which Eglantyne Jebb founded in 1919 (Beigbeder 1991, 187–188; Steinert 2008, 423), were analyzed, as well as those by people employed in its umbrella organization the Council of British Societies for Relief Abroad (COBSRA).^5^ COBSRA was founded in the summer of 1942 to coordinate the endeavors of the various British humanitarian associations responsible for providing care to those displaced as a consequence of the Second World War (Steinert 2008, 423–424, 434–435). Voices from this “new army of relief workers,” to borrow a phrase from Jessica Reinisch (2008, 371), provide vivid examples of child care in situations of displacement and also allow us to analyze the specific treatment of and attitudes towards displaced mothers on the part of certain aid organizations.

The second set of sources consulted were the personal case files held by the Arolsen Archives, the International Center on Nazi Persecution. Using the archive’s database,^6^ biographical case studies of single-mother families were mined to gain insights into the roles that the gender and family status of the applicants could play for their life trajectories. The case studies are based on the so-called “Care and Maintenance-Files (CM/1)” by the IRO which were accessed through the Digital Archive of the former International Tracing Service (ITS). The creation of these files reflects the institutional attempts to categorize and record the level of help needed by displaced people. The CM/1 files were distributed by the IRO from July 1947 in order to document which DPs were still dependent on and eligible to receive care from the organization (E-Guide Arolsen Archives 2023). Regarding the use of these files as historical sources, Huhn (2021, 11) observes: “Recent studies … have agreed that they [the CM/1 files] present narrations, strategies, and learning processes that DPs and refugees developed — collectively or alone — to negotiate with the IRO.” Besides revealing a lot about how international organizations categorized those uprooted populations in postwar Europe, they also allow us to retrieve the individual voices of those displaced. Therefore, the originality of this paper lies in its consideration of how displaced mothers were portrayed in sources created by relief workers, as well as its analysis of the strategies employed by these mothers and visible in the statements they made in their case files. The author of this article thereby shows that the postwar experience of displaced women cannot be understood by focusing solely on their particularly vulnerable position. It exemplifies that women and mothers, notwithstanding their difficult circumstances, could indeed navigate the postwar “migration regime” (Rass and Wolff 2018, 19), albeit with different outcomes.

Those displaced people assumed to be the most vulnerable in the context of postwar care and emigration planning were “‘unsupported mothers’ (widows/unmarried mothers) and their children” (Kevin and Agutter 2017, 554–555). The combined examination of the care mechanisms in place to support them and their children and of strategies employed by these mothers enriches the field of refugee studies by allowing it to “move beyond … stereotypical gender identities and ‘victim’ stories” of “vulnerable migrants” (Williams 2014, 452). When focusing on their situation, we cannot underestimate the importance of gender roles and hierarchies for the displacement experience. In this context, it is particularly interesting to examine the realms of care as well as the difficulties faced by mothers who were not “only” responsible for caring for their children and the “emotional labor associated with family migration” (Dellios 2018, 83) but also had to act as the head of the family.

## Care and Family as Categories of Analysis

Defining care and arguing for its importance as a category of analysis is essential if we want to understand the experiences of displaced mothers. Care in the postwar period “not only covered food, clothes, health care and ‘billeting,’ but also child welfare, occupational therapy, education, vocational training, and employment opportunities” (Cohen 2008, 441). Care organizations strongly promoted the idea of self-sufficiency of those displaced, as exemplified in a publication by UNRRA (1944) entitled “Helping the people to help themselves.” A relief worker expressed similar views in a letter to their COBSRA supervisor: “these people must learn to run themselves” (Peterkin 1948b, 1).

Historians should situate “care” as an important and often first point of contact with the institutional structures for organizing migration, repatriation, and resettlement from postwar Europe. The topics of care and caregiving are reflected quite extensively in the sources on the management of migration in the postwar period. Therefore files concerning the administration of health care dictate much of what we know about postwar migration regimes. They can provide insights into the everyday life of those displaced as well as their resettlement chances. In fact, the experience of displacement, as well as the possibilities for emigration, depended considerably on the individuals’ health and their requirements for medical care (Bienert and Kischlat 2014, 95–101). The medical examinations that were a compulsory part of every emigration process (Stieber 1997, 289–290) were conducted and recorded with little input from those displaced people wishing to emigrate. Illnesses or handicaps, but also other factors like an uncertain marital status, — for example, when the husband had disappeared, leaving his wife and family to wonder about his whereabouts — pregnancy or even being a single mother (Kay 2003, 158–160), could lead to the rejection of the applicants’ emigration requests.

If we think about postwar displacement and resettlement more broadly, family should be understood as another essential entity of analysis. Writing about the history of refugees coming to Australia, Dellios (2018, 68) notes that:

Most obviously, ‘family’ features in histories of mobility and refuge as a category of organisation by the State. This reality can be either a structural imposition or a justification for refugee rejection or acceptance, depending on the temporal and spatial context, and the racial and political histories of respective refugee groups and nations considering asylum seeker claims.

As a “category of organization,” family structures, as well as the marital status of refugees, played an important role in the management of postwar migration and displacement in occupied Austria. Instead of concentrating the analysis on “individual adults or nationally defined cohorts,” Dellios (2018, 79) advocates for “histories of groups and individuals with a variety of intersectional affiliations […] including that of family.” What is especially interesting for this article is her argument that introducing the family as a critical unit of examination is “complicating the passive victim stereotype often applied to refugees” (Dellios 2018, 80). A closer look at the role of family relations enables us to better understand the everyday lives of displaced single mothers, as will be shown on the following pages using regional and biographical case studies.

## Motherhood in Displacement

Health care measures within camps in postwar Austria were central to the postwar experiences of single mothers living there and allow insights into perceptions of gender and their importance for understanding concepts of postwar rehabilitation (Zahra 2011, 41, 56). The two regional contexts that act as the starting points for this paper's analysis of postwar experiences of displaced mothers are the towns Feffernitz and Kapfenberg in British-occupied Austria. In July 1948, there were 18 camps for displaced people in the British occupation zone of Austria, housing around 19,884 persons. Most of these refugee camps were located in Styria (Stieber 1997, 172). While the Styrian town Kapfenberg played an important role among the DP camps in British-occupied Austria since it was the location of a field office of the IRO (Stieber 1997, 289), Feffernitz camp stands out as the sole place of refuge for displaced “ethnic Germans” within the area of British-occupied Carinthia (Stieber 1997, 229). Feffernitz camp was created to house approximately 10,000 displaced people — many of them were “ethnic Germans” from Yugoslavia. However, in the months following the end of the war Feffernitz camp also housed Hungarian refugees. During its existence, between 3,000 and 3,500 people lived there (Stieber 1997, 228–229). In a relief worker's report, Feffernitz camp is described as having “all the usual features … associated with DP camps … workshops, schools, hospitals, a small library which gives untold pleasures, theatre, etc.” (Biddle 1948a, 1). Nonetheless, the condition of Feffernitz camp was quite poor, which was explained by the fact that young men left the camp during the day to work elsewhere: “The effect of this is … to leave the camp stripped of its youth … and to comprise only the aged, the infirm, the women with families and the children” (Biddle 1948a, 1).

Both the Feffernitz camp and the refugee camps in and around the Styrian town of Kapfenberg housed many so-called “ethnic Germans” (Stieber 1997, 228–231, 262–263). In the area surrounding Kapfenberg, the displaced people lived in seven different camps (SCF 1949, 2). During the Second World War, Kapfenberg played an important role in the armaments industry, as the so-called “Böhler Werke” were located there (Karner 2004, 57). Many forced laborers and prisoners of war had worked and lived in the barracks of the Böhler-Werke which were later used as DP camps (Stieber 1997, 262). After initially being under British and UNRRA administration (Stieber 1992, 450–453), the first DP camps in British-occupied Styria were handed over to the Austrian authorities in 1948. Only in 1950 were the other camps also placed under the control of Austrian authorities (Stieber 1992, 450, 468).

This paper argues that, the analysis of daily life in the camps for displaced people in Feffernitz and Kapfenberg provides insights into the experiences and problems that displaced mothers were confronted with. As Grossmann (2009, 204) has pointed out, the lives of DPs were characterized by the “rebuilding of properly gendered bodies and roles.” This went hand in hand with “attempts at reforming unmarried mothers” (Nowak 2019, 115). These attempts to reestablish “disrupted gender roles” (Nowak 2019, 114) can be exemplified by the fact that there were specific courses for displaced women to train them in skills classed as “female” and to learn how to be good mothers (Schein 1999; Nowak 2019, 122; Balint 2021, 62). This is also evident in reports on a DP camp in the Kapfenberg region documenting that: “handywork … is being encouraged/They [the women in the camp] do the most lovely traditional embroidery” (COBSRA 1948, 4). Likewise, in the camp in Feffernitz, relief work enforced gender norms as another report shows: “During the coming winter we plan to … have a sewing room for girls, and, if possible, apprenticeship classes for boys” (Biddle 1948a, 4).

One of this paper's goals, inspired by Dellios’s (2018, 83) appeal “to interrogate the categories imposed on refugee families,” is to highlight the importance of gendered hierarchies during displacement as well as during the emigration process. Displaced mothers often found themselves being judged harshly for their perceived parental inadequacies (Balint 2021, 88). This is especially true for single mothers whose case files allow insights into biased treatments by IRO officers due to their status as “unmarried.” Single mothers were often discriminated against by fellow camp inhabitants and in the procedures for resettlement abroad (Nowak 2019, 128–129). In reports by welfare workers, one can often find statements pointing out that DP women did not know how to act like mothers or were not fulfilling their role satisfactorily. A report on the hospitalization of sick children from the DP camp Feffernitz highlights that “it is very difficult to persuade the mothers sometimes to let the children come. They declare loudly that there is nothing wrong with the child and do not understand preventive medicine” (Biddle 1948b, 5). In a COBSRA report on a camp in Kapfenberg, inhabited by “ethnic German” Yugoslavs, it is stated that although children’s nutrition is mostly good, there are cases of sick children, which are attributed to “faulty feeding at home.” In order to deal with this problem, a so-called “mother and child clinic” was set up once a week. (Peterkin 1948a, 2). This criticism of DP mothers and the aid workers’ dedication to educating them has been interpreted by Balint (2021, 61–62) “as a response, in part, to the fear that women and girls had been profoundly defeminized by their wartime experiences and were in need of urgent domestic educating.” With regard to the gender biases apparent in the relief workers’ approach, Zahra writes: “at the same time that they idealized the family as the key to postwar rehabilitation, relief workers were skeptical about the ability of actual displaced parents (especially mothers) to care for their children” (2011, 56).

The living conditions in the various camps for displaced people were very different, which was also related to the purpose of the camps. Some of the refugee camps in Kapfenberg (Stieber 1997, 262) were a place of transit for DPs before they were resettled abroad. In a COBSRA report from 1948, one of the camps in the Kapfenberg region is therefore referred to as a “transit camp” for DPs moving on to work in the United Kingdom as part of the emigration scheme “Westward Ho!” (Allied Commission for Austria 1947, 36–37). From the Kapfenberg camps via Münster in Germany, many displaced people emigrated to the United Kingdom as so-called “European Volunteer Workers” (Mc Loughlin 2013, 253, 265–266). COBSRA workers in the “Westward Ho!” camp in Kapfenberg pointed out that they were “daily reminded that, owing to its transitory population, welfare is superfluous” (COBSRA 1948, 1). So, it seems, was privacy. Various reports point out the lack of intimate spaces within DP camps, as noted in April 1948 with regard to camp number seven in Kapfenberg: “This barrack camp is vastly over crowded [*sic*]. Sometimes as many as fifteen people in one room; in frequent cases unmarried single men or women are in a room with seven or eight married couples” (COBSRA 1948, 4). The cramped conditions and the lack of gendered spaces inside the barracks are also mentioned in other reports, which point out, for example, that: “10–15 people in one barrack room is … common place: families have been sorted out more or less, and attempts have been made to separate single men and women from married families, but this is not completed” (Peterkin 1948a, 2). An inspecting officer of DP camps in Austria expressed particular concern about the lack of separation of the sexes and the poor conditions of the camps and living quarters: “One finds all sorts of odd people living in the same room … there is acute over-crowding and a complete disregard of sexes. The sight of this enormous and almost terrifying squalor made me quite sick before my inspection ended” (Inspection report, December 1949, The National Archives of the UK, FO 1020/2836, quoted in: Stieber 1997, 268).

The care of displaced children and mother-child relations in general are recurring topics in the reports by relief workers in the Kapfenberg region, and expectant mothers are mentioned particularly often. Along with children, older people, and patients suffering from tuberculosis, they received larger food portions (Gardner-McTaggart 1948, 6). Their situation is described as often very difficult, with the COBSRA report from April 1948 mentioning “overwrought mothers of large families.” Having a small child in the postwar period could prove to be very bad for the personal health of mothers, who often found themselves alone in charge of childcare, as relief worker Clare McMurray mentioned in a report from October 1948:

2 months ago a woman in one of my camps asked if I could help in looking after her child — a baby girl of ten months. She had TB and had been in hospital for treatment. She was expecting another child and was due to go into hospital. The husband was out at work all day and there was no one to look after the child. I took the child to Eisenerz Kinderheim and the Doctor there took charge of her. The child was very much underweight and when screened it was found that she also had TB. (McMurray 1948, 8)

Not only pregnancy but also contagious conditions like tuberculosis or venereal diseases (Stieber 2005, 272) made women's displacement experiences particularly precarious. In relation to DP camps in occupied Germany, Haushofer has observed that in “British and American military correspondence” there was “a tendency to single out the female DP population as particularly prone to contracting VD” (2010, 4). Single women were especially likely to be suspected as carriers of sexually transmitted diseases. She points out that the British military government in occupied Germany asked UNRRA “to conduct compulsory VD examinations among all female DPs between the ages of 14 and 50 years who were not resident with their husbands” (2010, 4). Here, the biased treatment to which single displaced women were very often subjected becomes evident.

## Child Care in Displacement

In addition to caring for their own health, the everyday lives of DP mothers were dominated by child care responsibilities. Children were omnipresent in the daily life in the DP camps, as the annual report of Feffernitz shows: “One cannot be for very long within the precincts of Feffernitz without being very conscious of the children. They form the largest percentage of the population and are literally everywhere, on the roads riding on the trucks, playing in all the forbidden places and behaving as children will all the world over” (Biddle 1948a, 2). Children constituted almost half of the camp population in 1948 — the year in which the responsibility of the camp was given to the Austrian authorities (Stieber 1997, 228). The report also points out “the smallness of the children comparing with the general level of averages” (Biddle 1948a, 2). Of the approximately 3,000–3,500 camp inhabitants, around 600 children attended school, while 300 went to the camp kindergarten (Stieber 1997, 229–231). In the following passage of a relief worker's report, the high birth rate among the in-camp population is emphasized, as well as the important role that the maternity home played within the camp's infrastructure:

New babies are frequent in Feffernitz camp and there is a barrack set aside as the Maternity Home. It is very small, with only ten beds and is a miracle of whiteness, so much so, that it is an oasis in this otherwise sad camp. The midwife is a young Yugoslav woman of splendid character whose love for her work is all too apparent. She has managed to bring all the love and femininity which only such as she can. (Biddle 1948a, 3)

This description of the “femininity” of the midwife is another example for the emphasis on normative gender roles that become visible in postwar DP camps. In Feffernitz camp there also existed a facility for the care of children who had no parents to look after them: “There is a [m]other in charge of the house [ = the orphanage] and we also put there those children whose [m]others are temporarily unable to look after them … [due to] sickness or other domestic reasons” (Biddle 1948a, 3). As far as the help provided to large families was concerned, there were different attempts to offer the camp's youth and children educational, cultural, and sporting activities. One of these initiatives was the development of summer camps to rehabilitate children (COBSRA 1948, 3). For the children of the camp in Feffernitz, a summer camp was set up near Lake Faaker. Alongside displaced children, undernourished Austrian children were allowed to participate, thereby creating a contact space between the Austrian population and displaced people (Biddle 1948b, 4–5). With regard to child care, the annual report on Feffernitz further documents that there were conflicts in communicating with the mothers and that they often did not want to: “bring their children [to the maternity home in the camp]. They … wait until the child is really sick and has to be admitted to the hospital” (Biddle 1948a, 3).

Most of the children in Feffernitz camp were between six and 12 years old and thus exactly at school enrolment age. However, also in this camp, the standard of education suffered due to the desire to reduce camp expenses. While the children were supplied with food and health care, they lacked “possibilities for development internally” (Biddle 1948a, 4) as Biddle noted: “these children and young folks lead a most abnormal life — crowded together in barracks, which can by no stretch of imagination be called homes, and lacking every small refinement of privacy for toilet and everyday living. They can give no outlet to their fantasy or talents, and have little prospect as a future generation” (Biddle 1948a, 4). The reports by relief workers show that there was a “desperate shortage of school materials” (Biddle 1948a, 2) as well as of teachers in the DP camps, resulting in classes with sometimes 100 children per educator (Gardner-McTaggart 1948, 2–4). Furthermore, the displaced children were often an important “economic factor” for their parents. As there were “no facilities for them to train as apprentices within the [Feffernitz] camp nor outside in the Austrian district” (Biddle 1948a, 2) many children had to work as farm hands from an early age: “They are too poor ever too consider entering the Secondary School and there is little or no opportunity for them to enter the trade-schools … They do in fact become young workers on farms as soon as they are old enough and the difficulty is to persuade the parent to allow the child to attend the school for a little longer” (Biddle 1948a, 2). However, the same report also expresses a sense of hope for the future of those displaced children who “will show the world at large that in spite of living in DP camps … [they] have been educated up to the normal standards” (Biddle 1948a, 2).

## Leaving Austria Behind: Emigration Chances for Displaced Single Mothers

Being displaced and living in precarious conditions with few prospects in Austria led to some residents choosing to emigrate. The inhabitants of Feffernitz were no exception here, as this note by a relief worker suggests: “I have to explain at least twenty times per week that the receipts of affidavits are not the ‘open sesame’ to America and that the immigration laws have to be fulfilled” (Biddle 1948b, 7). The two biographical case studies selected for this paper exemplify attempts by two single-mother families to leave the DP camps in Kapfenberg and Feffernitz and their systems of care behind and resettle somewhere else. They provide new insights into the pathways of displaced families by broadening the perspective of care work for mothers and children in postwar DP camps to include an approach that makes the agency of displaced, unmarried women more visible. The case studies teach us that the opportunities for unmarried mothers and their families to emigrate were influenced by variable factors like their nationality, health, family status, marriage plans, and the age of their children, as well as their assessment by representatives of resettlement missions and IRO eligibility officers. Due to the lack of emigration opportunities and the fear of repatriation, some families were still living in the camp in Feffernitz in 1960 (Stieber 1997, 229).

By looking at the composition of families in postwar DP camps, we can see that they were characterized by separation and fragmentation. Relief workers had to assist divided families and were also responsible for reuniting them, as Biddle describes for the Feffernitz DP camp in Carinthia:

Among our camp inmates are many who are separated either from their children, or the children from their parents. We have also [m]others who have come from labour camps in Yugoslavia and whose children are held in that country. Where there appears a ghost of a chance that these families can be re-united we begin the enquiries through various Missions and HQ's [*sic*]. (Biddle 1948a, 4)

While family separation was a big problem, having children in postwar Europe could also be a significant challenge, especially when DPs tried to resettle somewhere else. Tara Zahra has pointed out that children living in DP camps in Germany “were … frequently abandoned … when their parents repatriated or emigrated” (Zahra 2011, 42). When thinking of family and gender in the context of migration, it must be emphasized that IRO eligibility officers judged women in relation to the senior male members of a family. Their classification as “dependents” is thus a recurring term in archival documents on postwar migration (Balint 2021, 60). This meant that, in general, if the head of the family was granted protected status by UNRRA or the IRO, this also applied to his wife and children. But there could also be negative consequences to this (Balint 2021, 62–64). If the husband was assumed to have been a Nazi collaborator and therefore not entitled to DP status and the IRO's help in emigration matters, neither were his wife or children (Balint 2021, 59). The reasons for this lie in the IRO's policy of not separating families (Balint 2021, 64–66), which has been interpreted as a reaction to the deliberate destruction of families by the National Socialists (Zahra 2011, 38). As Zahra pointed out, in the years following the end of the war, relief workers “sought to reestablish the sovereignty of families as much as the sovereignty of nations” (2011, 38).

However, in a postwar reality that was characterized by the separation from and loss of family members, single mothers were far from being the exception (Grossmann 2009, 230). A report by an IRO children's officer mentions that in a camp for “ethnic German” expellees in Feffernitz, 40 percent of all children were without a father (Biddle 1948b, 7). It has been pointed out that the standard organizational procedure of categorizing and registering displaced women as “dependents” also means that women’s experiences are less present in archival sources on postwar migration (Chopard 2023). This paper's focus on women whom relief organizations categorized as “heads” of the family therefore intends to ensure a gendered perspective, highlighting the experiences of and problems faced by women in DP camps in Austria and during the resettlement process. The case studies confirm the observation that the lives of displaced mothers and their children were dependent on the nationality attributed to the male head of the family by the IRO (Balint 2021, 60; Fitzpatrick 2021, 49; Williams 2014, 440). However, they also show that single displaced mothers used specific strategies to improve their chances of emigration.

The first case study to exemplify this is the story of Family D.^7^, who consisted of a female family head, her daughter (born in Feffernitz) and two older sons (Care and Maintenance File [CM/1] on Family D, November 1949, 3.2.1.3/80563676/ITS Digital Archive, Arolsen Archive*s*). As the handwritten notes on the interview of the female head of Family D. taken by the IRO officer suggest, the mother anticipated the problems that would be created by the nationality of her husband, who by that time was not living with the family. Her case file states: “She swears her husband is not a Volksdeutsche [ = ethnic German] — that he only registered as such in 1946 in order to get into Feffernitz Camp as there was no room in Kellerberg” (CM/1-file on Family D, November 1949, 3.2.1.3/80563676/ITS Digital Archive, Arolsen Archives). What becomes apparent here is that certain displaced people used their nationality or ethnic background in order to navigate the postwar migration regimes (Panagiotidis 2020, 181–182). This was a rather recurrent strategy among DPs and refugees, which Fitzpatrick has exemplified with her case studies of “White Russians” who had “falsified their identities and claimed other nationalities” (Fitzpatrick 2021, 44) in order to avoid being repatriated to the Soviet Union. The husband of Family D. used the category “ethnic German” in order to get a place in a specific camp largely inhabited by German-speaking refugees. However, it was precisely this strategy of her husband that later caused problems for the family, since people categorized as “ethnic Germans” had no entitlement to IRO care (Panagiotidis 2020, 181). Although the husband had identified himself previously as an “ethnic German,” his wife chose to do differently. She claimed instead that he was of Italian nationality, while the CM/1-file states that she and her children were of Romanian nationality (CM/1-file on Family D, November 1949, 3.2.1.3/80563676/ITS Digital Archive, Arolsen Archives) This is an interesting example of how the nationalities assigned to DPs from the outside, in this case by IRO personnel, often did not correspond with those they chose themselves. By asserting a different nationality for her husband, the mother tried to increase the family's chances of receiving support from the IRO and assistance in achieving their desire to resettle. However, declaring her husband an Italian citizen did not immediately benefit the emigration chances of Family D. After the mother had been interviewed, the CM/1-file records that there was “[n]o proof of husbands [*sic*] activities during war” and “no satisfactory proof that DP is Refugee” (CM/1-file on Family D, November 1949, 3.2.1.3/80563676/ITS Digital Archive, Arolsen Archives).

However, it was not only her husband's categorization as an “ethnic German” and the lack of information about his life during the war that created obstacles for the family's rehabilitation. Another reason for the dire situation the family found itself in was their separation from the father: “My husband left me 4 months ago and now lives in Germany.”^8^ Although he “wrote once after he reached Germany … since then she has heard nothing” (CM/1-file on Family D, November 1949, 3.2.1.3/80563676/ITS Digital Archive, Arolsen Archives). Consequently, the mother lived in Feffernitz camp alone with her three children. Her CM/1-file states that the family wished to emigrate, repatriation not having been an option for them: “I don’t want to find a life in Romania without my husband. … I am afraid of being arrested in Romania”^9^ (CM/1-file on Family D, November 1949, 3.2.1.3/80563676/ITS Digital Archive, Arolsen Archives). The mother must have been aware that, as a single woman with young children, she had few choices regarding emigration. In her answer to the question about the desired country of emigration, she only stated “where I am admitted.”^10^ In her file, it is further noted that the mother had relatives who already lived in Argentina. In 1950, the family was still registered in St Martin (Carinthia) — another DP camp and IRO field office (Stieber 1997, 289). However, a later search inquiry led by the daughter, who was trying to trace her father, indicates that the mother, together with her children, had indeed finally been able to emigrate to Australia: “in 1951 Mrs. … emigrated to Australia with her two sons by a former marriage and the baby [girl]” (TD File Inquiry 1960, 6.3.3.2/106483147/ITS Digital Archive, Arolsen Archives). The mother's decision to use her Italian-sounding maiden name instead of her married name when registering to embark for Australia also underscores her agency with regard to navigating the family's emigration process (Passenger List, 3.1.3.2/81680996/ITS Digital Archive, Arolsen Archives).

However, this case study also confirms the dependence of displaced women on the status of the “male head of the family” (Balint 2021, 61). It shows that the husband's nationality — whether by his own attribution or by that of the IRO — could have negative consequences for the whole family and result in them being assumed ineligible for IRO help with emigration. It also points to the fact that displaced women and children often found themselves separated from their husbands and fathers, either because they had perished, were not traceable, or because they had emigrated separately, leaving their families behind. This, of course, led to specific problems in terms of clarifying the women's marital status as well as difficulties with regard to balancing both child care and wage labor.

Despite this tendency for displaced single mothers to be ill-treated or perhaps precisely because of it, they tried to take control of their biography by choosing what to tell about themselves and what to leave out. Strategies like putting false names or birth dates on the application forms for assistance and emigration, becoming foreign or Austrian citizens by marriage, or using family networks abroad to emigrate more quickly could lead to better chances for a “successful” emigration. It is exactly this use of family connections that becomes clear in the second case study of this paper, that of Family U. The family consisted of a mother and her daughters, with whom she wanted to emigrate to Brazil, where the children's grandfather and her uncle were already living (Record of Interview, Family U. March 1950, 3.2.1.3/80855731/ITS Digital Archive, Arolsen Archives). The mother and her daughters had spent part of their lives in Austria in DP camp number two in Kapfenberg. Their case demonstrates how social networks could be used as an argument for a family's emigration plans. The mother of Family U. stressed: “I am afraid of persecution and I want to join my father in Brazil.”^11^ In her CM/1-file from June 1949 she stated the following: “My father … has been accepted by the IRO and has already left for Brazil”^12^ (CM/1-file on Family U, June 1949, 3.2.1.3/80855728/ITS Digital Archive, Arolsen Archives). Her remarks could be interpreted as attempts to improve her chances of receiving help from the IRO by stating that two close family members — both male — had already successfully emigrated.

In her CM/1-file, the applicant's nationality is described as “Yugoslav (Croat).” In October 1945, she had fled from Yugoslavia to Austria, since her father had already been expelled from there by the partisans. The records of her interview by the IRO document that she and her oldest daughter were both in good health. The mother is described as “rather shy … but with character. A good mother, poor but clean and healthy looking” (Record of Interview, Family U. March 1950, 3.2.1.3/80855731/ITS Digital Archive, Arolsen Archives). Another common feature of the CM/1-files dealing with female heads of families becomes evident here, namely the examination of the external appearance of the displaced women as proof of their eligibility for care and resettlement. The role of gender expectations in the determination of the DPs’ status should not be underestimated, as Balint points out when stating that IRO files “also hint at the ways in which the men of the board responded to women who performed their femininity in acceptable ways” (Balint 2021, 66).

The interview records on Family U. further mention that the female family head planned to marry the father of her children, who was within the mandate of the IRO. Together they wanted to emigrate, as an entry in her case file from August 1950 states: “Unmarried mother with 2 children, one child only 2 months old and therefore not suitable for emigration, would also like to marry an invalid (right arm amputated) and emigrate together with him”^13^(Beratungsdienst in Österreich, August 1950, 3.2.1.3/80855729/ITS Digital Archive, Arolsen Archives). Their emigration plans were unsuccessful, at least in part due to the young age of the child. The observation that “her husband-to-be earns very little”^14^ (Beratungsdienst in Österreich, August 1950, 3.2.1.3/80855729/ITS Digital Archive, Arolsen Archives) — probably due to his physical handicap — might also have played a role here. Furthermore, the interview records mention her status as an “unmarried mother” and the couple's “matrimonial plans” as “reasons why [the] family group … cannot be resettled at this time” (Record of Interview, Family U. March 1950, 3.2.1.3/80855731/ITS Digital Archive, Arolsen Archives). No records could be found showing that the family emigrated from Austria in the postwar years. There are, however, documents indicating that the family still lived in a Carinthian DP camp at the end of the 1950s (Refugee/Migrant Registration, Family U. August 1958, 3.2.2.1/81395877/ITS Digital Archive, Arolsen Archives).

As demonstrated by this case study, the agency of displaced mothers in postwar Austria was influenced by several factors, such as the fact that they had a young child or their choice of spouse. The story of Family U. further exemplifies that although displaced single mothers could exercise a certain agency in navigating the emigration procedures, their applications for resettlement were often unsuccessful. This meant that many women continued to rely on the care infrastructures provided in Austria. It was not only their own biographical background that influenced their chances of emigration but also their marital status and the nationality and health of their husbands. Even if they were living separate from them or were not even married yet, their trajectories were influenced by the biographies of the male “heads of the family.” However, despite these constraints, displaced single mothers tried to fulfill their desire to emigrate by using certain strategies, such as dissociating themselves from their husbands by declaring a different nationality and using their maiden names, or by capitalizing on other social networks.

## Conclusion

The experiences of displaced mothers living in Austria were varied, as were their possibilities for navigating the “migration regimes” in place. In light of the individual stories that have been the focus of the previous pages, this paper argues that the diverse experiences of displaced single mothers can best be explored through a multilevel approach that considers both practices at the institutional level — i.e., measures to improve the particularly vulnerable position of displaced mothers in Austrian DP camps and the expectations of relief workers towards them, — as well as the strategies of these women themselves. This paper shows that displaced women in postwar Austria faced diverse problems resulting from their marital status and childcare obligations. Their lives in the postwar period were largely shaped by their specific needs for care as well as by gender imbalances within the process of obtaining a resettlement opportunity. The gendered analysis of the relief workers’ reports and the CM/1-files proved fruitful in this paper's endeavor to highlight both areas of vulnerability and agency for displaced single mothers. Only by considering these two dimensions can the postwar situation of displaced women be adequately described. Instead of focusing solely on the particularly vulnerable position single mothers found themselves in, the paper also highlights strategies that they employed to take their future and that of their children into their own hands. With regard to the two camps in British-occupied Austria which were more closely examined — Kapfenberg (Styria) and Feffernitz (Carinthia) — different areas of care specifically provided to displaced mothers and their children were described, ranging from nutrition and health care to education and vocational training. Hereby the focus of analysis lay on the everyday experiences and difficulties faced by displaced mothers and their children. In this context, the reports by aid workers from the COBSRA and SCF provided insights into dire living conditions within the camps as well as attempts to improve them. While these enabled the analysis of what Zahra called “gendered rehabilitation” (2011, 58) measures, the examination of the case studies from the Arolsen Archives made the heterogeneous life paths and different forms of agency exerted by displaced women more visible. It was found that although single mothers had a unique position within the camp — in the sense that they were entitled to more care measures, such as special feeding programs — their precarious situation often became an obstacle to their resettlement efforts which forced them to come up with new strategies to succeed in emigrating. A closer look at the institutionalization of care in the portrayed camps, as well as a focus on the personal files of single mothers as main applicants for emigration, revealed that they were not only cared for by relief workers, but that they strategically negotiated their emigration options in an effort to take control over their own futures.

## Notes

1 The results depicted in this paper form part of the author's broader research on this topic, within the project “Norms, Regulation and Refugee Agency: Negotiating the Migration Regimes” at the University of Vienna, funded by the Austrian Science Fund (FWF) and the German Research Foundation (DFG). The paper is based on the author's ongoing research for her dissertation project as well as on her presentation at the international conference “Families in Transit,” organized by Prof. Dr. Sarah Cramsey at Leiden University in September 2022. More information on the project at the University of Vienna and Osnabrück University can be found on the project website: https://displacement-and-migration-regimes.univie.ac.at/

2 In order to better reflect the postwar realities of this particular group of DPs, the author of this article uses a broad definition of the term “single mother,” which also encompasses parents who lived separately or families where the father was absent.

3 In German as well as in English texts the word “Volksdeutsche” is frequently used.

4 The author is aware of the problem of referring to DPs or “ethnic German” refugees and expellees as two “groups” since they comprised people with very diverse national and religious backgrounds.

5 I would like to give my special thanks to Katherine Wositzky, who kindly made these reports available to me and who published a very interesting website on her mother’s activities as a relief worker in DP camps in postwar Europe: https://www.displacedpersoncampcollection.org/

6 Arolsen Archive, https://arolsen-archives.org/suchen-erkunden/suche-online-archiv/

7 In order to safeguard the privacy of those families described and their next generations the author has obscured the individuals’ names.

8 Translation from German by the author of this paper. In the original: “Mein Mann hat mich seit vier Monaten verlassen und lebt jetzt in Deutschland.”

9 Translation from German by the author of this paper*.* In the original: “ohne meinen Mann moechte [ich] in Rumänien keine Lebensmöglichkeit finden. … Ich fuerchte mich in Rumänien verhaftet zu sein [*sic*].”

10 Translation from German by the author of this paper. In the original: “Wo ich aufgenommen werde.”

11 Translation from German by the author of this paper. In the original: “ich habe Angst vor Verfolgungen und will zu dem Vater nach Brasilien.“

12 Translation from German by the author of this paper. In the original: “Mein Vater … ist von der IRO angenommen [worden] und … schon nach Brasilien abgereist.”

13 Translation from German by the author of this paper. In the original: “Uneheliche Mutter mit 2 Kindern, ein Kind erst 2 Monate alt daher nicht geeignet fuer ein Auswanderung [*sic*], moechte ausserdem heiraten, einen Invaliden (rechter Arm amputiert) und gemeinsam mit ihm auswandern.”

14 Translation from German by the author of this paper. In the original: *“*ihr angehender Ehemann nur wenig verdient”.
